# Whole-Body MRI-Derived Adipose Tissue Characterization and Relationship to Pulmonary Function Impairment

**DOI:** 10.3390/tomography8020046

**Published:** 2022-02-27

**Authors:** Ricarda von Krüchten, Susanne Rospleszcz, Roberto Lorbeer, Dunja Hasic, Annette Peters, Fabian Bamberg, Holger Schulz, Stefan Karrasch, Christopher L. Schlett

**Affiliations:** 1 Department of Diagnostic and Interventional Radiology, Medical Center—University of Freiburg, Faculty of Medicine, University of Freiburg, 79106 Freiburg, Germany; ricarda.kruechten@uniklinik-freiburg.de (R.v.K.); dunja.hasic@uniklinik-freiburg.de (D.H.); fabian.bamberg@uniklinik-freiburg.de (F.B.); 2 Institute of Epidemiology, Helmholtz Zentrum München, German Research Center for Environmental Health, Ingolstädter Landstraße 1, 85764 Neuherberg, Germany; susanne.rospleszcz@helmholtz-muenchen.de (S.R.); peters@helmholtz-muenchen.de (A.P.); scicon@t-online.de (H.S.); 3 Institute for Medical Information Processing, Biometry and Epidemiology, Medical Faculty, Ludwig-Maximilians-University, 81377 Munich, Germany; stefan.karrasch@med.uni-muenchen.de; 4 Department of Radiology, Ludwig-Maximilians-University Hospital, 80336 Munich, Germany; roberto.lorbeer@med.uni-muenchen.de; 5 German Center for Diabetes Research, München-Neuherberg, 85764 Neuherberg, Germany; 6 Institute and Outpatient Clinic for Occupational, Social and Environmental Medicine, Inner City Clinic, University Hospital of Munich, Ludwig-Maximilians-Universität, 80336 Munich, Germany; 7 Comprehensive Pneumology Center Munich, Member of the German Center for Lung Research, Munich-Neuherberg, 85764 Neuherberg, Germany

**Keywords:** respiratory function tests, lung diseases, abdominal fat depots, hepatic fat content, magnetic resonance imaging

## Abstract

*Background*: Specification of adipose tissues by whole-body magnetic resonance imaging (MRI) was performed and related to pulmonary function parameters in a population-based cohort. *Methods*: 203 study participants underwent whole-body MRI and pulmonary function tests as part of the KORA (Cooperative Health Research in the Augsburg Region) MRI study. Both visceral adipose tissue (VAT) and subcutaneous adipose tissue (SAT) were derived from the T1-Dixon sequence, and hepatic adipose tissue from the proton density fat fraction (PDFF_hepatic_). Associations between adipose tissue parameters and spirometric indices such as forced vital capacity (FVC), forced expiratory volume in 1 s (FEV1) and Tiffeneau-index (FEV1/FVC) were examined using multivariate linear regression analysis excluding cofounding effects of other clinical parameters. *Results*: VAT (β = −0.13, *p* = 0.03) and SAT (β = −0.26, *p* < 0.001), but not PDFF_hepatic_ were inversely associated with FEV1, while VAT (β = −0.27, *p* < 0.001), SAT (β = −0.41, *p* < 0.001), and PDFF_hepatic_ (β = −0.17, *p* = 0.002) were inversely associated with FVC. PDFF_hepatic_ was directly associated with the Tiffeneau index (β = 2.46, *p* < 0.001). *Conclusions*: In the adjusted linear regression model, VAT was inversely associated with all measured spirometric parameters, while PDFF_hepatic_ revealed the strongest association with the Tiffeneau index. Non-invasive adipose tissue quantification measurements might serve as novel biomarkers for respiratory impairment.

## 1. Introduction

Obesity, especially abdominal obesity and the related metabolic syndrome, is a major global health problem. It is an important risk factor for type II diabetes and cardiovascular disease, increased morbidity and mortality, and escalating health care costs [1].

Non-invasive diagnostic investigation of different types of adipose tissue is ongoing and promising. Abdominal obesity includes subcutaneous (SAT), visceral adipose tissue (VAT) and more specifically hepatic fat by using proton density fat fraction (PDFF_hepatic_).

In contrast to VAT and SAT, specifically PDFF_hepatic_ was associated to a higher risk for pro-inflammatory mediators and cardiometabolic disease [2]. Obesity is differentiated from non-alcoholic fatty liver disease (NAFLD) and increasing knowledge about the anatomical body fat distribution enables more effective metabolic risk stratification. VAT more than SAT induces metabolic changes such as insulin resistance, accelerated atherosclerosis and pro-inflammation accelerating cardiometabolic disease development [3].

Prior studies showed an association of diabetes and metabolic syndrome parameters with impaired pulmonary function [4,5]. VAT is a major risk factor for diabetes and the metabolic syndrome [6] and abdominal adiposity is related to respiratory impairment [7,8,9]. The underlying pathophysiology is multifactorial, and not only the mechanical ventilation restriction due to higher diaphragm resistance should be considered [10]. Few studies showed that increased VAT, most likely due to endocrine-mediated factors, predisposes to a pro-inflammatory state associated with bronchopulmonary dysfunction [11]. Moreover, NAFLD has been shown to be associated with reduced forced expiratory volume in 1 s (FEV1) and forced vital capacity (FVC) [12].

Body fat distribution can be reliably determined by clinical examination (body mass index, waist-to-hip ratio), computed tomography (CT), dual-energy radiography absorptiometry, abdominal ultrasound [13], and magnetic resonance imaging (MRI) enabling high precision measurement of hepatic and visceral adipose tissues [14,15]. Methods for abdominal obesity imaging include relaxation-based (T1 fast spin sequence), phase sensitive and fat selective MRI. Complementing diagnostic modalities for the metabolic syndrome, MRI is radiation-free and has been employed in multiple prospective cohort studies [16].

Body composition measures may be novel prognostic biomarkers that influence clinical outcome of patients with COVID-19. CT- derived VAT/SAT ratio and intramuscular adipose tissue were related to adverse outcomes independent of other established risk factors in COVID-19 patients [17]. In another study higher VAT measurements by CT were observed in COVID-19 patients that required hospitalization [18].

The objective of this population-based cross-sectional study was to assess MRI-derived local abdominal fat depots in relation to pulmonary function in a healthy cohort study from the western population.

## 2. Materials and Methods

### 2.1. Study Design and Population

The Cooperative Health Research in the Region of Augsburg (KORA) F4 cohort study represents a broad sample from a general population in the region of Augsburg (Germany). The study recruited 1851 subjects aged between 25–74 years [19]. A magnetic resonance imaging scan (3 Tesla) was acquired in the FF4 follow-up MRI sub-study and participants were examined between June 2013 and September 2014 at the KORA study center. Subjects aged 39–74 were selected for an MRI whole-body scan (*n* = 400).

Exclusion criteria were: (1) contraindications to MRI, gadolinium administration and (2) a known cardiovascular disease defined as validated/self-reported stroke, myocardial infarction or prior cardiac revascularization. Due to incomplete MRI data and/or inadequate image quality, 22 out of 400 subjects were excluded. Spirometry results were available in 226 subjects from the FF4 cohort excluding subjects with a history of respiratory disease such as asthma, COPD, and ever-smokers [20].

The KORA MRI cohort complied with the Helsinki declaration [21] on human research and was approved by the Institutional Research Ethics Board of the Medical Faculty of Ludwig-Maximilian University Munich. All participants provided informed consent for study participation.

### 2.2. Clinical Characteristics

Detailed information on clinical characteristics was collected in all study participants. Subjects were classified as smokers if they had smoked at least one cigarette per day in the year prior to the study and number of pack years were documented.

Diabetic state was defined as followed: 

*Normoglycemic*: 2-h serum glucose concentration determined by oral glucose tolerance test (OGTT) below 140 mg/dL, and a fasting glucose level below 110 mg/dL. 

*Prediabetes*: Normal fasting glucose concentration and a 2-h serum glucose concentration, as determined by OGTT, ranging between 140 and 200 mg/dL; and/or an impaired fasting glucose concentration, as defined by fasting glucose levels between 110 and 125 mg/dL, and a normal 2-h serum glucose concentration.

*Diabetes* (2-h serum glucose concentration as determined by OGTT that was >200 mg/dL and/or a fasting glucose level that was >125 mg/dL).

Further laboratory testing determined cholesterol (high density lipoprotein HDL, low density lipoprotein LDL), triglycerides and high-sensitive C-reactive protein (hsCRP) concentrations in all participants. In addition, physical activity, hypertension (systolic blood pressure > 140 mmHg, diastolic blood pressure > 90 mmHg, or antihypertensive treatment), lipid lowering medication, cholesterol status, and alcohol consumption (no, moderate, heavy) were registered using the standardized KORA MRI protocol [19,22].

Spirometry was performed in line with the American Thoracic Society and European Respiratory Society recommendations [23]. Flow-volume curves were acquired using a pneumotachograph-type spirometer (MasterScope, Jaeger, Hoechberg, Germany). The participants performed at least three, and up to eight spirometric maneuvers to obtain a minimum of two acceptable and reproducible readings. More details have been reported earlier [20]. Obstructive lung disease was defined as FEV1/FVC < LLN, with the “lower limit of normal” (LLN) calculated according to the Global Lung Function Initiative (GLI) 2012 equations [24].

### 2.3. Whole-Body MR Imaging

Whole-body MRI scans were performed with a 3 Tesla MR system (Magnetom Skyra, Siemens AG, Healthcare Sector, Erlangen, Germany). The protocol comprised sequences covering the body from neck to below hip for tissue/organ quantification. Participants were scanned in the supine position. An 18-channel body coil combined with the table-mounted spine matrix coil was used for each scan.

For analysis of the body adipose tissue volume, a 2-point Dixon T1 sequence in submaximal inspiration breath hold and an acquisition time of 15 s was used. Slice thickness (ST) was 1.7 mm, coronal acquired, including a field of view (FOV) of 488 × 716, a matrix of 256 × 256, a repetition time (TR) of 4.06 ms and an echo time (TE) of 1.26 ms. As a reliable measure of hepatic fat content, the PDFF_hepatic_ was determined as proton density fat fraction automatically reconstructed by using a Multi-echo Dixon VIBE sequence in a single breath hold with a ST of 4 mm, coronal acquired, including a FOV of 393 × 450, a matrix of 256 × 179, a TR of 8.9 ms and six echo TE of 1.23 ms; 2.46 ms; 3.69 ms; 4.92 ms; 6.15 ms; 7.38 ms [19]. All image analysis was performed by blinded independent experienced readers who were unaware of the clinical characteristics of study subjects on dedicated off-line workstations.

Based on the volume-interpolated, three-dimensional fat images from the 2-point-Dixon sequence, a fat-selective tomogram was calculated (slice thickness 5 mm in 5 mm increments). An in-house algorithm based on Matlab R2013a (MathWorks, Natick, MA, USA) was used to semi-automatically quantify total adipose tissue from the femoral head to the cardiac apex, VAT from the femoral head to the diaphragm, and SAT from the femoral head to the cardiac apex [25]. The algorithm automatically segmented the tissue components VAT and SAT based on fuzzy clustering and orthonormal snakes within approximately 2 min per data set. Thresholds were defined at 50% of the maximum fat signal automatically detected in each slice. All segmentations were manually adjusted, if necessary, due to imperfections at the borderline between VAT and SAT [26].

PDFF_hepatic_ was quantified using estimated proton-density fat fraction by accounting for the confounding effects of T2* decay and the spectral complexity of fat [15]. Three regions of interest with a size of 30 × 30 m^2^ were manually placed in the right (segment VIII) and left (segment II) liver lobe on each of the three slices at the level of the portal vein, avoiding large vessels and surrounding extrahepatic tissue [15,27]. PDFF_hepatic_ was defined as the average of the right and left lobe measurements.

### 2.4. Statistical Analysis

Clinical characteristics, spirometric parameters, and MR-derived adipose tissue measures were presented as arithmetic means with standard deviation (SD) for continuous variables. If the visual inspection of the variable’s distribution revealed severe non-normality, the variable was presented as median with interquartile range

Differences in these variables between participants with and without obstructive lung disease were evaluated by *t*-test or Wilcoxon-test, as appropriate. Categorical variables are reported as counts and percentages and differences were assessed by χ^2^ test. Associations of MR derived adipose tissue measures (VAT, SAT and PDFF_hepatic_) as exposure variables and spirometric parameters (FEV1, FVC, FEV1/FVC) as outcome variables were assessed by linear regression models. PDFF_hepatic_ was log-transformed before modelling and all exposure variables were standardized to mean 0 and SD 1. Resulting estimates thus represent changes in spirometric parameters for one standard deviation of adipose tissue variables. All models were adjusted for age, gender and body surface area (BSA), and in a second step additionally adjusted for smoking status (never, former, current), pack-years, diabetes status and presence of hypertension. In a third step, the model was additionally adjusted for physical activity (no vs. yes). As sensitivity analyses, selected models were adjusted for hsCRP, presence of obesity as defined by body mass index (BMI) >30, or alcohol consumption (no, moderate, heavy). Statistical analyses were performed using R version 3.6 (R Core Team, R Foundation for Statistical Computing, Vienna, Austria).

## 3. Results

**Figure 1:**
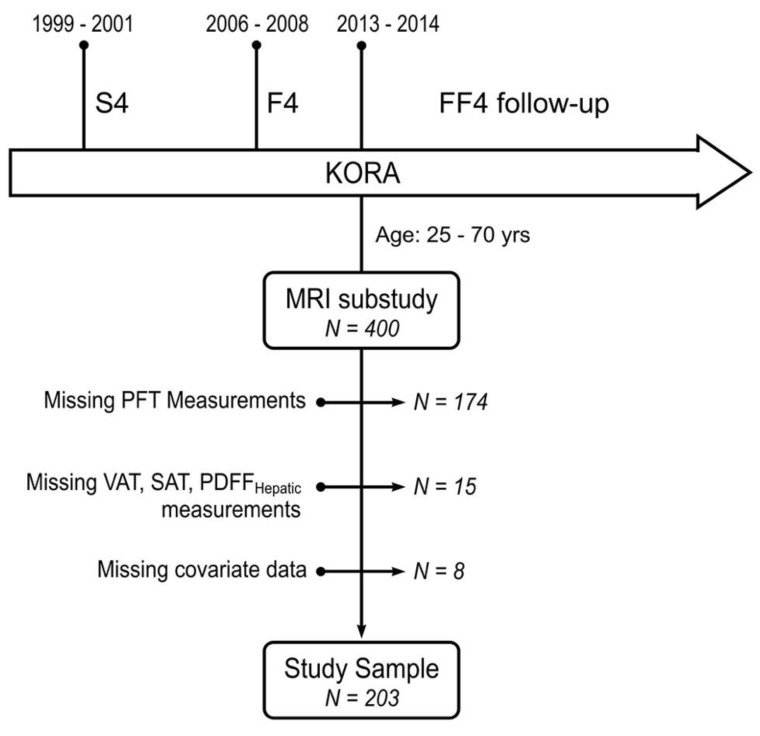
Study inclusion scheme. From a total of 400 subjects who underwent whole-body MRI, 197 subjects had to be excluded due to missing pulmonary function test measurements, missing VAT, SAT, PDFF_hepatic_ measurements, and missing covariate data. VAT = visceral adipose tissue; SAT = subcutaneous adipose tissue; PDFF = proton density fat fraction; PFT = pulmonary function test.

A total of 400 subjects underwent whole-body MR imaging, with 174 subjects excluded due to missing lung function data, 15 subjects were excluded due to poor image quality and inappropriate VAT, SAT, PDFF_hepatic_ measurements, and eight subjects due to missing covariate data. Hence complete data and results were obtained from a total of 203 subjects (Figure 1). Mean age of study subjects was 58.0 ± 5.8 years, with slightly more males (57.6%) than females, 39% former smokers, 22% current smokers and 39% non-smokers. Further characterizations of the study sample are shown in Table 1.

The total abdominal adipose tissue was 12.9 ± 5.1 L, consisting of VAT, which totaled 4.8 ± 2.7 L, and SAT totaling 8.1 ± 3.4 L. Median PDFF_hepatic_ was 4.7% [IQR: 2.9–13.1%].

Cholesterol, triglyceride and hsCRP concentrations did not differ between the subgroups (Table 1 and Table 2).

In 23 subjects obstructive lung disease (11.3%) was detected with a mean FEV1/FVC of 59.7 ± 6.9% and FEV1 2.6 ± 0.8 L/s (Table 1). PDFF_hepatic_ differed significantly between subjects with and without obstructive lung disease (3.9% vs. 5.4%; *p* = 0.04; Table 2).

### Spirometric Parameters in Association with MR-Derived Adipose Tissue

FEV1 and FVC were inversely associated to all three fat depots after adjusting simple statistical models for age, sex, and BSA (Table 3). After multiple adjustment for potential confounders including smoking, diabetes and hypertension, VAT and SAT remained inversely associated with FEV1, while the association for PDFF_hepatic_ became insignificant (*p* = 0.14). For FVC, all three fat depots remained inversely associated after adjustments (all *p* < 0.001). Additional adjustment for physical activity did not affect effect estimates (Table 3). More importantly, these associations were not significantly affected by additional adjustment for obesity as measured by BMI (Table 3).

In contrast, the Tiffeneau index (FEV1/FVC) was positively associated with PDFF_hepatic_ in the simple (*p* < 0.001) and the fully adjusted model (*p* < 0.001). For VAT, the Tiffeneau index showed a positive association in the adjusted model, while SAT was never associated to the Tiffeneau index (Table 3).

In subjects with no or moderate alcohol consumption, a total of 61 subjects (41%) had a PDFF_hepatic_ of >6.4% which equals to the presence of non-alcoholic steatohepatitis (NASH) [24]. Additional adjustment for alcohol consumption (no, moderate, heavy) did not alter the association of continuous measure of PDFF_hepatic_ with FVC (ß = −0.24, *p* < 0.001 vs. ß = −0.22, *p* < 0.001 with and without additional adjustment for alcohol consumption based on the full model).

## 4. Discussion

Whole-body MRI-derived adipose tissue types were associated with functional lung parameters. The following key findings from our population-based cohort study in a total of 203 subjects can be concluded: (1) By using whole-body MRI adipose tissue measurements only VAT was inversely associated with all measured spirometric parameters, while (2) PDFF_hepatic_ revealed the strongest association with the Tiffeneau Index.

Consistent with our findings, Thijs et al. also reported an inverse association between MRI-derived VAT and FEV1 as well as FVC [9]. Additionally, we observed an inverse correlation between SAT with FEV1 and FVC, while in their study SAT was not correlated. This could be explained by their focus on subjects with a high waist circumference, compared to our sample which was based on a cohort study from the general population.

In several studies, including a population-based study of 21,550 subjects in the UK, hip-ratio methods for assessing abdominal obesity showed that both FEV1 and FVC were linearly and inversely related to abdominal obesity [28]. However, body mass index (BMI) and waist circumference measurements were generally less accurate surrogate markers for visceral fat estimation. Additionally, fat distribution may be more strongly associated with pulmonary function than weight or BMI [29]. In a French population-based study comprising 121,965 subjects, abdominal obesity measured by waist circumference was a predictor of reduced FEV1 and FVC, independent of BMI [30].

Another population-based study of 1145 subjects by Choe et al. using computed tomography for abdominal fat evaluation and spirometry, showed that total adipose tissue (TAT; comprising VAT and SAT) as well as VAT were inversely associated with FEV1 and FVC [31], consistent with greater accuracy for radiologically-obtained fat measurements. Over a follow-up period over 3 years, TAT and VAT were linked to decreasing FEV1 and FVC, while decreasing TAT and VAT were linked to increasing FEV1 and FVC, supporting a significant correlation with weight reduction. In contrast to our results, Choe et al. reported no association with SAT, even though both techniques are able to differentiate between and quantify SAT and VAT depots. It has to be considered, that SAT in general functions rather as an indicator for general fat.

In our study we used whole-body MRI to determine SAT and VAT volumetrically, which is a precise, safe and radiation-free imaging modality for measuring fat compartments as a favorable alternative to CT for quantification [32]. We further determined PDFF_hepatic_ as PDFF in MRI which is currently considered as the non-invasive standard complementing other diagnostic modalities. We found an inverse association between MRI-derived PDFF of the liver and FVC, independent of the analyzed confounders and more importantly, independent of obesity. Our findings are consistent with previous studies in Asian and American adults that used mainly ultrasonography for the evaluation of NAFLD [33,34,35]; however, they reported an association to FEV1 which we could not reproduce. This could be explained by limited statistical power and low sample size, or method comparability (ultrasound vs. MRI) and race differences, respectively. Even in a sub analysis considering alcohol intake, FEV1 was not associated with PDFF_hepatic_ in our sample. It remains unclear why PDFF_hepatic_ was elevated in the group of non-obstructive lung disease individuals in our study.

Recently, reduced lung function was even described as a risk factor for incident NAFLD in a large middle-aged Korean cohort [36]. The underlying mechanisms still remain unclear. Other authors report that insulin resistance plays a role, as it is closely associated with both NAFLD [37] and reduced lung function [38]. However, after adjustment for diabetes in our models, the fat depots remained associated with impaired lung function independent of diabetic status.

One strength of our study is the use of an advanced MRI technology 3-Tesla generation, which includes the most advanced imaging modality to-date with well-defined imaging protocol, image processing, and detailed information on the health condition of the study population. However, our study contains limitations which should be considered. First, our study is a cross-sectional analysis design and therefore does not allow to assess relationships between local abdominal fat depots and pulmonary function over time. Second, our sample size was relatively small and as it was based on a cohort from the general population, it may have limited generalizability. Third, respiratory diseases such as COPD are multifactorial and a number of risk factors or comorbidities such as cardiovascular disease and heart failure were not considered in this study. Forth, while physical activity is an essential parameter for observational health studies, accurate assessment remains challenging. Moreover, although the measurement of liver fat represents an average of six different sizes with a sufficiently large ROI, it must be considered that liver fatty degeneration can also occur heterogeneously. Finally, though MRI is more favorable to ultrasonography or CT regarding assessment of liver fat [39], it has limitations such as clinical contraindications, higher costs and availability of the described MRI sequences.

Therefore, future machine learning algorithms might include standardized measurements of fat depots in available MRI-scans to provide useful additional data for prevention of obesity-related comorbidities including pulmonary impairment. This could apply to subjects with obstructive as well as restrictive ventilatory patterns who would benefit from weight management interventions. Furthermore, body composition measurements might be used as novel biomarkers to predict the clinical course in COVID-19 or other respiratory diseases. Goehler et al. concluded that VAT volumes ≥ 100 cm^2^ were associated with a greater risk of severe disease or death in COVID-19 [40].

In conditions where high MRI-derived VAT volumes are diagnosed pulmonological function tests could be advised. VAT more than SAT is a convincing MRI measurement parameter to anticipate decreased respiratory capacity.

## 5. Conclusions

In conclusion, VAT was reversely associated with all measured spirometry parameters. Visceral obesity was associated with decreased lung function and should be prevented in asymptomatic subjects to prevent pulmonary and metabolic disease progression. This study encourages to investigate body composition measurements as novel biomarkers for respiratory diseases.

## Figures and Tables

**Table 1:** Characteristics of the study sample.

	Whole Sample	Subjects with OLD	Subjects without OLD	*p*-Value
Total amount	203	23	180	
Age, years	58.0 ± 5.8	56.6 ± 6.1	58.1 ± 5.7	0.22
Men	117 (57.6%)	16 (69.6%)	101 (56.1%)	0.32
Height, cm	171.4 ± 10.0	173.8 ± 11.9	171.1 ± 9.7	0.22
BMI, kg/m^2^	28.0 ± 4.4	27.0 ± 5.0	28.2 ± 4.3	0.21
Body Surface Area, m^2^	1.9 ± 0.2	2.0 ± 0.2	1.9 ± 0.2	0.86
Smoking				0.53
never-smoker	79 (38.9%)	7 (30.4%)	72 (40.0%)	
ex-smoker	79 (38.9%)	9 (39.1%)	70 (38.9%)	
smoker	45 (22.2%)	7 (30.4%)	38 (21.1%)	
Pack-years (only ex-smoker and smoker)	20.2 ± 20.4	25.5 ± 19.3	19.5 ± 20.5	0.27
Glycemic status				0.82
normoglycemic	122 (60.1%)	13 (56.5%)	109 (60.6%)	
prediabetes	53 (26.1%)	6 (26.1%)	47 (26.1%)	
diabetes	28 (13.8%)	4 (17.4%)	24 (13.3%)	
Physically active	124 (61.1%)	15 (65.2%)	109 (60.6%)	0.84
Hypertension	73 (36.0%)	8 (34.8%)	65 (36.1%)	1.00
Antihypertensive Medication	55 (27.1%)	5 (21.7%)	50 (27.8%)	0.72
Lipid-lowering Medication	25 (12.3%)	2 (8.7%)	23 (12.8%)	0.75
Total Cholesterol, mg/dL	221.8 ± 37.4	219.5 ± 44.9	222.1 ± 36.4	0.76
HDL Cholesterol, mg/dL	62.5 ± 17.7	58.5 ± 15.6	63.0 ± 17.9	0.24
LDL Cholesterol, mg/dL	141.9 ± 34.3	140.5 ± 40.2	142.1 ± 33.6	0.84
Triglycerides, mg/dL	137.5 ± 90.9	154.6 ± 126.9	135.3 ± 85.4	0.34
Alcohol consumption, g/d	21.7 ± 27.1	19.4 ± 22.3	22.0 ± 27.7	0.66
Pulmonary Function Test				
FEV1/FVC, %	74.8 ± 7.7	59.7 ± 6.9	76.7 ± 5.2	<0.001
FEV1, L/s	3.1 ± 0.8	2.6 ± 0.8	3.2 ± 0.8	0.001
FVC, L	4.2 ± 1.0	4.4 ± 1.1	4.2 ± 1.0	0.39

Subjects were stratified in “presence of obstructive lung disease” and “no obstructive lung disease”. Obstructive lung disease was defined as FEV1/FVC < LLN. The values represent mean ± standard deviation (SD) or frequency along with percentage. *p*-values from *t*-test or Χ^2^ test, as appropriate, between group with OLD and without OLD. BMI = body mass index; HDL Cholesterol = High-density lipoprotein cholesterol; LDL = Low-density lipoprotein cholesterol, FEV1 = Forced expiratory volume in 1 s; FVC = Forced vital capacity; LLN = lower limit of normal; OLD = obstructive lung disease.

**Table 2:** Comparison of MR derived adipose tissue and hsCRP between the subgroups.

	Whole Sample	Presence of OLD	Absence of OLD	*p*-Value
Visceral adipose tissue, L	4.8 ± 2.7	4.2 ± 2.8	4.8 ± 2.7	0.31
Subcutaneous adipose tissue, L	8.1 ± 3.4	7.4 ± 3.8	8.2 ± 3.3	0.26
Total abdominal adipose tissue, L	12.9 ± 5.1	11.6 ± 5.8	13.1 ± 5.0	0.20
PDFF_hepatic_, % (median[Q1, Q3])	4.7 [2.9, 13.1]	3.9 [1.9, 6.1]	5.4 [2.9, 14.5]	0.04
hsCRP, mg/L (median[Q1, Q3])	1.1 [0.6, 2.4]	1.0 [0.7, 4.0]	1.1 [0.6, 2.2]	0.53

Obstructive lung disease was defined as FEV1/FVC < LLN. Data are presented as means and standard deviation, unless otherwise indicated. *p*-value for comparison between group with OLD and without OLD by either *t*-test or Wilcoxon test, as appropriate. L= liter, PDFF_hepatic_ = proton density fat fraction; hsCRP = high-sensitive c-reactive protein; OLD = obstructive lung disease.

**Table 3:** Associations of MR derived adipose tissue with outcome FEV1, FVC and FEV1/FVC.

		FEV1			FVC			FEV1/FVC		
	Model	β	95%CI	*p*	β	95%CI	*p*	β	95%CI	*p*
VAT										
	simple	−0.23	[−0.34, −0.13]	<0.001	−0.38	[−0.49, −0.26]	<0.001	1.15	[−0.36, 2.65]	0.13
	adjusted	−0.19	[−0.30, −0.08]	<0.001	−0.35	[−0.48, −0.23]	<0.001	1.71	[0.09, 3.33]	0.04
	+physAct	−0.19	[−0.30, −0.08]	0.001	−0.34	[−0.47, −0.22]	<0.001	1.68	[0.04, 3.32]	0.045
	+obesity	−0.13	[−0.25, −0.02]	0.03	−0.27	[−0.40, −0.14]	<0.001	1.50	[−0.22, 3.23]	0.09
SAT										
	simple	−0.31	[−0.41, −0.20]	<0.001	−0.49	[−0.60, −0.37]	<0.001	1.25	[−0.34, 2.84]	0.12
	adjusted	−0.31	[−0.42, −0.21]	<0.001	−0.49	[−0.60, −0.37]	<0.001	1.20	[−0.45, 2.84]	0.15
	+physAct	−0.48	[−0.60, −0.36]	<0.001	−0.31	[−0.42, −0.20]	<0.001	1.17	[−0.49, 2.83]	0.166
	+obesity	−0.26	[−0.39, −0.14]	<0.001	−0.41	[−0.55, −0.28]	<0.001	0.84	[−1.06, 2.75]	0.38
PDFF_hepatic_										
	simple	−0.09	[−0.18, −0.01]	0.03	−0.24	[−0.33, −0.15]	<0.001	2.16	[1.01, 3.32]	<0.001
	adjusted	−0.07	[−0.17, 0.02]	0.14	−0.22	[−0.33, −0.11]	<0.001	2.55	[1.23, 3.86]	<0.001
	+physAct	−0.07	[−0.16, 0.03]	0.180	−0.21	[−0.32, −0.11]	<0.001	2.54	[1.21, 3.87]	<0.001
	+obesity	−0.03	[−0.12, 0.06]	0.52	−0.17	[−0.27, −0.06]	0.002	2.46	[1.11, 3.81]	<0.001

The simple model was adjusted for age, gender and BSA. The full model included age, gender, BSA, smoking, diabetes, hypertension, pack-years. The full model was additionally adjusted for physical activity, and obesity. β-coefficients are from linear regression and denote the change in spirometric outcomes per one standard deviation of the adipose tissue; CI, confidence interval. FEV1 = Forced expiratory volume in 1 s; FVC = Forced vital capacity; VAT = visceral adipose tissue; SAT = subcutaneous adipose tissue; PDFF_hepatic_ = proton density fat fraction.

## Data Availability

The informed consent given by KORA study participants does not cover data posting in public databases. However, data are available upon request by means of a project agreement. Requests should be sent to kora.passt@helmholtz-muenchen.de and are subject to approval by the KORA Board.

## References

[1] Fox C.S., Massaro J.M., Hoffmann U., Pou K.M., Maurovich-Horvat P., Liu C.-Y., Vasan R.S., Murabito J.M., Meigs J.B., Cupples L.A. (2007). Abdominal visceral and subcutaneous adipose tissue compartments: Association with metabolic risk factors in the Framingham Heart Study. Circulation.

[2] Cioffi C.E., Narayan K.M.V., Liu K., Uppal K., Jones D.P., Tran V., Yu T., Alvarez J.A., Bellissimo M.P., Maner-Smith K.M. (2020). Hepatic fat is s stronger correlate of key clinical and molecular abnormalities than visceral abdominal subcutaneous fat in youth. BMJ Open Diabetes Res. Care.

[3] Hamdy O., Porramatikul S., Al-Ozairi E. (2006). Metabolic obesity: The paradox between visceral and subcutaneous fat. Curr. Diabetes Rev..

[4] Chen W.-L., Wang C.-C., Wu L.-W., Kao T.-W., Chan J.Y.-H., Chen Y.-J., Yang Y.-H., Chang Y.-W., Peng T.-C. (2014). Relationship between lung function and metabolic syndrome. PLoS ONE.

[5] Paek Y.-J., Jung K.-S., Hwang Y.-I., Lee K.-S., Lee D.R., Lee J.U. (2010). Association between low pulmonary function and metabolic risk factors in Korean adults: The Korean National Health and Nutrition Survey. Metabolism.

[6] Oka R., Kobayasi J., Inazu A., Yagi K., Miyamoto S., Sakurai M., Nakamura K., Miura K., Nakagawa H., Yamagishi M. (2010). Contribution of visceral adiposity and insulin resistance to metabolic risk factors in Japanese men. Metabolism.

[7] Chen Y., Breithaupt K., Muhajarine N. (2000). Occurrence of chronic obstructive pulmonary disease among Canadians and sex-related risk factors. J. Clin. Epidemiol..

[8] Chen Y., Dales R., Tang M., Krewski D. (2002). Obesity may increase the incidence of asthma in women but not in men: Longitudinal observations from the Canadian National Population Health Surveys. Am. J. Epidemiol..

[9] Thijs W., Dehnavi R.A., Hiemstra P.S., de Roos A., Melissant C.F., Janssen K., Tamsma J.T., Rabe K.F. (2014). Association of lung function measurements and visceral fat in men with metabolic syndrome. Respir. Med..

[10] Unterborn J. (2001). Pulmonary function testing in obesity, pregnancy, and extremes of body habitus. Clin. Chest Med..

[11] Gan W.Q., Man S.F., Senthilselvan A., Sin D.D. (2004). Association between chronic obstructive pulmonary disease and systemic inflammation: A systematic review and a meta-analysis. Thorax.

[12] Mantovani A., Lonardo A., Vinco G., Zoppini G., Lippi G., Bonora E., Loomba R., Tilg H., Byrne C.D., Fabbri L. (2019). Association between non-alcoholic fatty liver disease and decreased lung function in adults: A systematic review and meta-analysis. Diabetes Metab..

[13] Gruzdeva O., Borodkina D., Uchasova E., Dyleva Y., Barbarash O. (2018). Localization of fat depots and cardiovascular risk. Lipids Health Dis..

[14] Yokoo T., Serai S.D., Pirasteh A., Bashir M.R., Hamilton G., Hernando D., Hu H.H., Hetterich H., Kühn J.-P., Kukuk G.M. (2018). Linearity, Bias, and Precision of Hepatic Proton Density Fat Fraction Measurements by Using MR Imaging: A Meta-Analysis. Radiology.

[15] Hetterich H., Bayerl C., Peters A., Heier M., Linkohr B., Meisinger C., Auweter S., Kannengießer S.A.R., Kramer H., Ertl-Wagner B. (2016). Feasibility of a three-step magnetic resonance imaging approach for the assessment of hepatic steatosis in an asymptomatic study population. Eur. Radiol..

[16] Kim S.R., Lerman L.O. (2018). Diagnostic imaging in the management of patients with metabolic syndrome. Transl. Res..

[17] Bunnell K.M., Thaweethai T., Buckless C., Shinnick D.J., Torriani M., Foulkes A.S., Bredella M.A. (2021). Body composition predictors of outcome in patients with COVID-19. Int J. Obes..

[18] Chandarana H., Dane B., Mikheev A., Taffel M.T., Feng Y., Rusinek H. (2021). Visceral adipose tissue in patients with COVID-19: Risk stratification for severity. Abdom. Radiol..

[19] Bamberg F., Hetterich H., Rospleszcz S., Lorbeer R., Auweter S.D., Schlett C.L., Schafnitzel A., Bayerl C., Schindler A., Saam T. (2017). Subclinical Disease Burden as Assessed by Whole-Body MRI in Subjects with Prediabetes, Subjects with Diabetes, and Normal Control Subjects from the General Population: The KORA-MRI Study. Diabetes.

[20] Karrasch S., Flexeder C., Behr J., Holle R., Huber R.M., Jörres R.A., Nowak D., Peters A., Wichmann H.-E., Heinrich J. (2013). Spirometric reference values for advanced age from a South german population. Respiration.

[21] (2013). World Medical Association Declaration of Helsinki: Ethical principles for medical research involving human subjects. JAMA.

[22] Mueller J., Karrasch S., Lorbeer R., Ivanovska T., Pomschar A., Kunz W.G., von Krüchten R., Peters A., Bamberg F., Schulz H. (2019). Automated MR-based lung volume segmentation in population-based whole-body MR imaging: Correlation with clinical characteristics, pulmonary function testing and obstructive lung disease. Eur. Radiol..

[23] Miller M.R., Hankinson J., Brusasco V., Burgos F., Casaburi R., Coates A., Crapo R., Enright P., van der Grinten C.P.M., Gustafsson P. (2005). Standardisation of spirometry. Eur. Respir. J..

[24] Quanjer P.H., Stanojevic S., Cole T.J., Baur X., Hall G.L., Culver B.H., Enright P.L., Hankinson J.L., Ip M.S.M., Zheng J. (2012). Multi-ethnic reference values for spirometry for the 3-95-year age range: The global lung function 2012 equations. Eur. Respir. J..

[25] Würslin C., Machann J., Rempp H., Claussen C., Yang B., Schick F. (2010). Topography mapping of whole body adipose tissue using A fully automated and standardized procedure. J. Magn. Reson. Imaging.

[26] Storz C., Heber S.D., Rospleszcz S., Machann J., Sellner S., Nikolaou K., Lorbeer R., Gatidis S., Elser S., Peters A. (2018). The role of visceral and subcutaneous adipose tissue measurements and their ratio by magnetic resonance imaging in subjects with prediabetes, diabetes and healthy controls from a general population without cardiovascular disease. Br. J. Radiol..

[27] Lorbeer R., Bayerl C., Auweter S., Rospleszcz S., Lieb W., Meisinger C., Heier M., Peters A., Bamberg F., Hetterich H. (2017). Association between MRI-derived hepatic fat fraction and blood pressure in participants without history of cardiovascular disease. J. Hypertens..

[28] Canoy D., Luben R., Welch A., Bingham S., Wareham N., Day N., Khaw K.T. (2004). Abdominal obesity and respiratory function in men and women in the EPIC-Norfolk Study, United Kingdom. Am. J. Epidemiol..

[29] Ochs-Balcom H.M., Grant B.J., Muti P., Sempos C.T., Freudenheim J.L., Trevisan M., Cassano P.A., Iacoviello L., Schünemann H.J. (2006). Pulmonary function and abdominal adiposity in the general population. Chest.

[30] Leone N., Courbon D., Thomas F., Bean K., Jégo B., Leynaert B., Guize L., Zureik M. (2009). Lung function impairment and metabolic syndrome: The critical role of abdominal obesity. Am. J. Respir. Crit. Care Med..

[31] Choe E.K., Kang H.Y., Lee Y., Choi S.H., Kim H.J., Kim J.S. (2018). The longitudinal association between changes in lung function and changes in abdominal visceral obesity in Korean non-smokers. PLoS ONE.

[32] Klopfenstein B.J., Kim M.S., Krisky C.M., Szumowski J., Rooney W.D., Purnell J.Q. (2012). Comparison of 3 T MRI and CT for the measurement of visceral and subcutaneous adipose tissue in humans. Br. J. Radiol..

[33] Moon S.W., Kim S.Y., Jung J.Y., Kang Y.A., Park M.S., Kim Y.S., Chang J., Ro J.S., Lee Y.-H., Lee S.H. (2018). Relationship between obstructive lung disease and non-alcoholic fatty liver disease in the Korean population: Korea National Health and Nutrition Examination Survey, 2007–2010. Int. J. Chron. Obstruct. Pulmon. Dis..

[34] Jung D.-H., Shim J.-Y., Lee H.-R., Moon B.-S., Park B.-J., Lee Y.J. (2012). Relationship between non-alcoholic fatty liver disease and pulmonary function. Intern. Med. J..

[35] Peng T.-C., Kao T.-W., Wu L.-W., Chen Y.-J., Chang Y.-W., Wang C.-C., Tsao Y.-T., Chen W.-L. (2015). Association between Pulmonary Function and Nonalcoholic Fatty Liver Disease in the NHANES III Study. Medicine.

[36] Song J.-U., Jang Y., Lim S.-Y., Ryu S., Song W.J., Byrne C.D., Sung K.-C. (2019). Decreased lung function is associated with risk of developing non-alcoholic fatty liver disease: A longitudinal cohort study. PLoS ONE.

[37] Takamura T., Misu H., Ota T., Kaneko S. (2012). Fatty liver as a consequence and cause of insulin resistance: Lessons from type 2 diabetic liver. Endocr. J..

[38] Lazarus R., Sparrow D., Weiss S.T. (1998). Baseline ventilatory function predicts the development of higher levels of fasting insulin and fasting insulin resistance index: The Normative Aging Study. Eur. Respir. J..

[39] Reeder S.B., Cruite I., Hamilton G., Sirlin C.B. (2011). Quantitative Assessment of Liver Fat with Magnetic Resonance Imaging and Spectroscopy. J. Magn. Reson. Imaging.

[40] Goehler A., Hsu T.-M.H., Seiglie J.A., Siedner M.J., Lo J., Triant V., Hsu J., Foulkes A., Bassett I., Khorasani R. (2021). Visceral adiposity and severe COVID-19 disease: Application of an artificial intelligence algorithm to improve clinical risk prediction. Open Forum Infect. Dis..

